
JOURNAL INFORMATION
==============================
NLM Title Abbreviation: Tex Med J (Austin)
NLM Journal ID: 21130590R

Texas Medical Journal

ISSN: 0892-8495
Publisher: Biomedical Journal Digitization Project

ARTICLE INFORMATION
==============================
PMCID: PMC9642583
Article version: 1
Subjects: Books and Magazines

Books and Magazines

Print publication date: 1912 Jul
Volume: 28
Issue: 1
First page: 37
Last page: 38
License: This work is free of known copyright restrictions. For more information, please see PMC Back Issue Digitization.
License URL: https://creativecommons.org/publicdomain/mark/1.0/

==============================

THE CHAS. H. PHILLIPS CHEMICAL CO.
NEW YORK and LONDON

PHILLIPS' MILK OF MAGNESIA
"THE PERFECT ANTACID"

SUPERIOR TO LIME WATER, BICARBONATE OF SODA, CHALK, ETC.
for correcting Hyperacid conditions—local or systemic.
Vehicle for Salicylates, Iodids, Balsams, etc.

TRADE MARK

PHILLIPS' REFINED CAMPHOR
PHILLIPS' WHEAT PHOSPHATES
PHILLIPS' DIGESTIBLE COCOA
PHILLIPS' PHOSPHO-MURIATE OF QUININE
PHILLIPS' SYRUP OF WHEAT PHOSPHATES
PHILLIPS' EMULSION OF COD LIVER OIL

2. The needle must be of wide bore.

3. Care should be taken when the piston is being replaced, the glass barrel being held firmly in its metallic casing with the left hand. The author advises those wishing to try this method to practice it with some simple oil.

4. There should be no hurry in the mixing, as the whole of the salvarsan will mix up with the oil. He has had no caking of the salvarsan in the syringe.—*Charlotte Medical Journal.*

### Books and Magazines.

Practical Medicine, Series 1912. Year Book Publishers, Chicago. Price, $10 for the set of ten—each year.

This series covers the entire field of medicine and surgery, and is about half-way between the monthly journal and the text-books. It is about a year ahead of the latter. It is published primarily for the general practitioner, at the same time the arrangement in several volumes enables those interested in special subjects to buy only the parts they desire. We have received Volumes 1 and 2

of the 1912 series—General Medicine, Billings-Salisbury, price $1.50; General Surgery, Murphy, price $2.00.

---

LABORATORY METHODS. WITH SPECIAL REFERENCE TO THE NEEDS OF THE GENERAL PRACTITIONER. By B. G. R. Williams, M. D., and E. G. C. Williams, M. D., with an introduction by Victor C. Vaughan, M. D., LL. D. 43 engravings. Price, $2.00. St. Louis, 1912. C. V. Mosby Co,

This manual seems to me to go at it right. Most authors assume a knowledge of elementary work students do not possess, and hence they can not understand. Beginners want A, B, C; want to be told *how* to go to work and what they need. This book supplies this. The publishers say:

"The authors have striven to make practicality the chief purpose of the work. They have striven to give the student and the country practitioner those methods the application of which will get results in laboratory work. They have striven to remove from the minds of the general practitioner the idea that they can not do laboratory work themselves, because they have no expensive laboratory equipment, and, as Dr. Vaughan says in his introduction, have not the opportunity to work in marble halls."

---

A ligature should not be placed on the carotid too near the bifurcation lest the cloth which forms shall not have sufficient surface to which to adhere and become detached and swept to the brain.—*American Journal of Surgery.*

---

**A publication that can hold its best advertising patronage more than a quarter of a century must be a good advertising medium. The reason: The Doctors read the ads; they are important. Mention the "Red Back" when writing to advertisers.**

### Publisher's Department.

IN THE gastro-intestinal derangement of young children, the profession will find Elixir Maltopepsine, Tilden's, a most excellent curative and palatable prescription. Free samples to the profession upon application to The Tilden Company, either New Lebanon, N. Y., or St. Louis, Mo.

---

A SUGGESTION IN TETANUS.—The physician who has ever faced the horrors of tetanus and has seen his ministration go for naught,
